# One-year quality of life and functional outcomes after robotic diaphragm plication for unilateral paralysis: A retrospective cohort analysis

**DOI:** 10.1016/j.xjon.2026.102084

**Published:** 2026-08-26

**Authors:** Philip Baum, Raffaela Griffo, Matthias Grott, Henrike Deissner, Alessio Campisi, Mavi Schellenberg, Felix Herth, Jacopo Lenzi, Konstantin Uttinger, Hauke Winter, Martin E. Eichhorn

**Affiliations:** a Department of Thoracic Surgery, Thoraxklinik, Heidelberg University Hospital, Heidelberg, Germany; b Department of Pulmonology, Thoraxklinik at Heidelberg University Hospital, Heidelberg, Germany; c Department of Biomedical and Neuromotor Sciences, University of Bologna, Bologna, Italy; d Department of General, Abdominal, Transplant and Thoracic Surgery, University Hospital Frankfurt, Frankfurt am Main, Germany; e Translational Lung Research Center Heidelberg (TLRC), German Center for Lung Research (DZL), Heidelberg, Germany

**Keywords:** diaphragm plication, health-related quality of life, pulmonary function, robotic-assisted thoracic surgery, unilateral diaphragm paralysis

## Abstract

**Objectives:**

Unilateral diaphragm paralysis is a rare thoracic condition that can significantly impair respiratory function and quality of life. This retrospective study aims to evaluate the long-term functional and quality of life outcomes after robotic-assisted thoracoscopic diaphragm plication.

**Methods:**

Between 2019 and 2024, 82 patients with symptomatic unilateral diaphragm paralysis were included in a prospectively maintained database at a single institution. All patients underwent robotic-assisted thoracoscopic diaphragm plication. Quality of life was assessed using the Diaphragmatic Paralysis Questionnaire, and lung function was evaluated by body plethysmography and spirometry in the upright position. Postoperative complications were graded according to the Clavien–Dindo classification.

**Results:**

Quality of life improved significantly across all Diaphragmatic Paralysis Questionnaire domains at 3 months and 1 year (all *P* < .001). Overall improvements were maintained over time, although the respiratory domain showed a modest change between 3 months and 1 year (*P* = .013). Forced expiratory volume in 1 second and forced vital capacity increased significantly at both follow-up time points (all *P* < .001). No significant changes were observed in arterial blood gas parameters (partial pressure of oxygen and partial pressure of carbon dioxide). Diffusing capacity of the lung for carbon monoxide showed a modest improvement at 3 months (*P* = .009), which was not sustained at 1 year (*P* = .066). Within 90 days, 6 patients (7.3%) developed Clavien–Dindo grade III or greater complications, whereas pleural effusion (grade I) was the most frequent event.

**Conclusions:**

This retrospective analysis of a prospectively collected cohort suggests that robotic-assisted thoracoscopic diaphragm plication is associated with improved long-term quality of life and pulmonary function in patients with symptomatic unilateral diaphragm paralysis.

## Graphical Abstract

**Figure:**
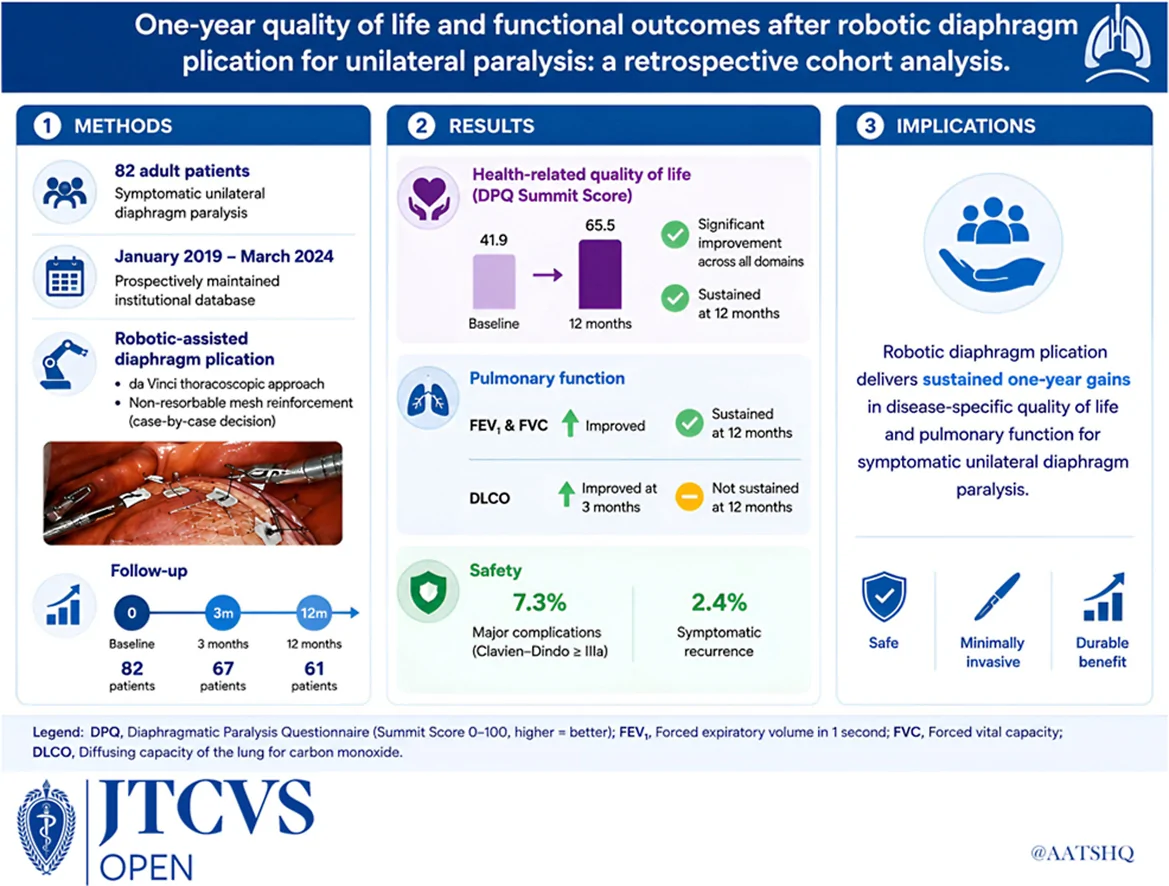

**Figure:**
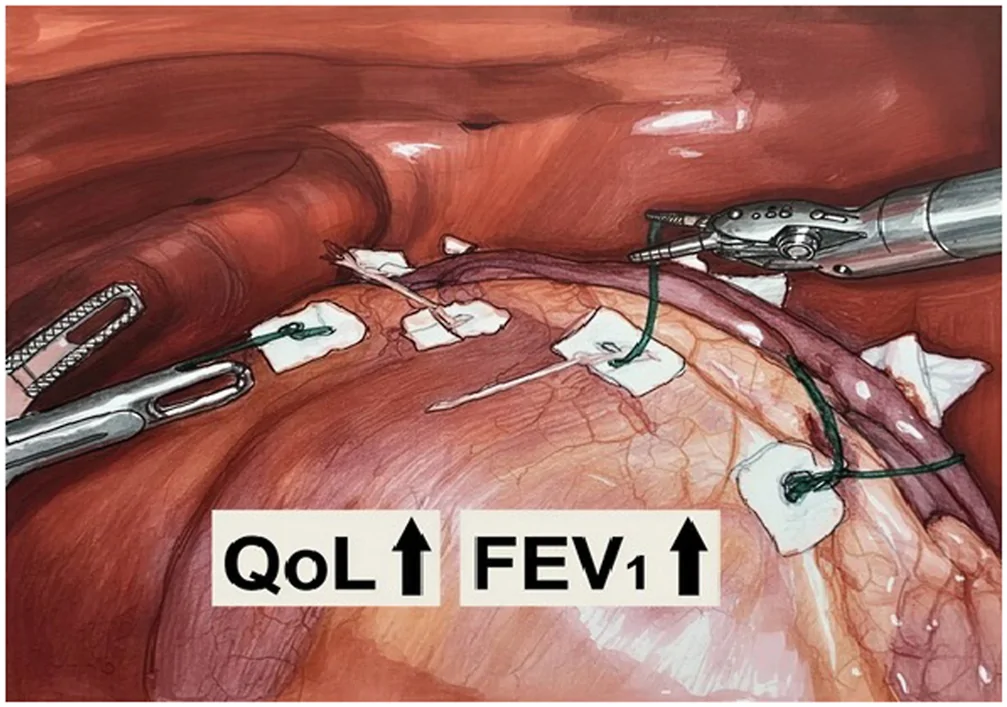
Robotic diaphragm plication improves quality of life and lung function up to 1 year.

**Central Message**

Robotic diaphragm plication is associated with sustained improvements in pulmonary function and disease-specific quality of life up to 1 year in patients with unilateral diaphragm paralysis.

**Perspective**

Unilateral diaphragm paralysis is an under-recognized condition with limited data on patient-centered outcomes. This study represents one of the largest retrospective robotic cohorts and provides the first longitudinal evaluation of disease-specific quality of life, suggesting a potential role of robotic diaphragm plication as a treatment option with sustained clinical benefit.

Unilateral diaphragm paralysis is a rare but clinically significant thoracic disorder that may cause dyspnea, orthopnea, and recurrent respiratory infections.^1^^,^^2^ The potential causes include traumatic, iatrogenic, infectious, and idiopathic etiologies, with idiopathic cases accounting for up to 50% of all patients.^1^ Diagnosis is based on imaging and functional assessment, including fluoroscopy,^3^ diaphragmatic ultrasound,^4^ and pulmonary function tests. Because spontaneous recovery may occur and reversible causes must be excluded, surgery is usually deferred for at least 6 to 12 months when the etiology is uncertain.^1^^,^^5^^,^^6^

Diaphragm plication aims to flatten and immobilize the paralyzed hemidiaphragm at its maximal inspiratory position, thereby relieving lung compression and improving pulmonary mechanics. The technique has evolved from open thoracotomy to minimally invasive video-assisted thoracoscopic surgery and, more recently, robotic-assisted approaches.^7^^,^^8^ Although the procedure is well established in routine clinical practice, there is a lack of detailed prospective studies on the long-term effects of the treatment on patients' quality of life. Previous studies have evaluated quality of life after diaphragm plication using general or respiratory-specific instruments such as the 36-item Short Form (SF-36), the St George's Respiratory Questionnaire (SGRQ), and the Medical Research Council (MRC) dyspnea scale, demonstrating improvements in symptoms and functional status after surgery.^9^, ^10^, ^11^ However, disease-specific assessment of quality of life in this patient population remains limited. Because existing evidence on the subject largely consists of small-scale reports (Table E1), there is a need for a more comprehensive understanding of surgical treatment and the quality of life afterward. So far, no research has systematically examined changes in health-related quality of life (HRQL) before and after robotic diaphragm plication using a validated questionnaire for this disease. The newly developed Diaphragmatic Paralysis Questionnaire (DPQ) was created by an expert panel of pulmonary physicians experienced in diagnosing and treating these patients, and it addresses the respiratory, physical, psychological, and social aspects of diaphragm paralysis.^12^ We present a retrospective analysis of a prospectively collected monocentric cohort of patients undergoing robotic-assisted thoracoscopic diaphragm plication, focusing on both objective pulmonary function and patient-reported quality of life outcomes.

## Patients and Methods

This was a retrospective observational study based on a prospectively collected institutional cohort of patients who underwent robotic-assisted diaphragm plication between January 2019 and March 2024 at the Department of Thoracic Surgery, Thoraxklinik at Heidelberg University Hospital. The database and diagnostic algorithm for patients with diaphragmatic paralysis were established in 2019. The study was conducted in accordance with the Declaration of Helsinki and followed the Strengthening the Reporting of Observational Studies in Epidemiology guidelines for observational studies (Table E2). All patients provided written informed consent for the use of clinical data for research purposes at hospital admission, and the study was approved by the University of Heidelberg Ethics Committee (Ethics Approval No. S-089/2018 on March 1, 2018). Symptomatic (dyspnea/orthopnea) adult patients with unilateral diaphragm paralysis confirmed by imaging and functional testing were eligible. Patients were initially evaluated by a pulmonologist and subsequently referred for surgical consultation if symptoms persisted for more than 6 to 12 months despite conservative management. Diagnostic evaluation included chest computed tomography, fluoroscopy, lung function assessment, magnetic resonance imaging of the cervical spine, and administration of the DPQ.^12^ Exclusion criteria included bilateral paralysis, neuromuscular disease, or incomplete data at baseline. Quality of life was assessed using the DPQ, a disease-specific validated instrument designed for patients with diaphragmatic dysfunction. The DPQ comprises multiple domains, including physical, respiratory, psychological, and social functioning, which are summarized into a global score (Summit Score). Scores range from 0 to 100, with higher values indicating better quality of life and fewer symptoms. The DPQ has been validated and shown to reliably capture patient-reported outcomes in this population, with good internal consistency and construct validity.^12^

All procedures were performed minimally invasive under general anesthesia with double-lumen endotracheal intubation and the patient placed in the semi-flexed lateral decubitus position. A da Vinci X Surgical System (Intuitive Surgical, Inc) was used. The operation used a 3-arm, 4-port approach: one 8-mm camera port, two 8-mm robotic working ports, and one 12-mm assistant port (Kii Advanced Fixation CFS22, Applied Medical).

The camera port was introduced along the fifth or sixth intercostal space anterior to the scapular tip, followed by carbon dioxide insufflation at 12 mm Hg. Under direct vision, the 2 working ports were placed 8 to 10 cm apart anteriorly and posteriorly, and the assistant port was positioned near the costal arch. The robot was docked from a 15° to 30° posterior–caudal angle toward the diaphragm. A needle driver and bipolar grasper were used for suturing.

Interrupted horizontal mattress U-sutures with nonresorbable material (Ethibond ExcelTM 2/MO-6; Ethicon) were placed in the dorso-ventral direction, beginning medially and progressing laterally. Polytetrafluoroethylene pledgets (Santec Medical) were used to reinforce each knot. Carbon dioxide insufflation was intermittently released to assess diaphragm tension and exclude herniation. Typically, 5 to 8 U-sutures were applied. After implementation of the surgical technique, monofilament polypropylene mesh (Bard Mesh; BD) was placed in an onlay fashion and fixed with interrupted stitches. At the end of the surgical procedure, an intercostal nerve blockage under endoscopic guidance was performed using ropivacaine (7.5 mg/mL, 20 mL), and a 24 Charriere chest tube was inserted and connected to a portable chest drainage system (Thopaz, Medela).

Patients followed a standardized postoperative pain management protocol and early respiratory physiotherapy to promote lung reexpansion. The chest tube was typically removed once full expansion and minimal drainage (<100 mL/24 hours) were confirmed.

Follow-up evaluations were scheduled at 1, 3, and 12 months postoperatively. At each visit, upright lung function testing, an x-ray, and DPQ assessment were performed. Comorbidities from administrative data were quantified using the Charlson Comorbidity Index based on International Classification of Diseases–coded comorbidities without age adjustment.^13^^,^^14^ Postoperative complications were graded according to the Clavien–Dindo classification. Data were collected in a prospective database (FileMakerProAdvanced 18, Claris).

Continuous variables were summarized as means and SDs or as medians and interquartile ranges, depending on the distribution of the data. Categorical variables were reported as absolute frequencies and percentages. To facilitate a comprehensive evaluation, paired *t* tests and Wilcoxon signed-rank tests were used to systematically assess the continuous outcomes at various time points, including baseline, 3-month, and 12-month marks. A total of 17 patients did not undergo their follow-up examination within 12 months of the initial visit. A complete-case analysis was performed to explore associations between preoperative variables and quality of life 1 year after surgery, as measured by the DPQ Summit Score. Predictors of 1-year quality of life were analyzed using linear regression models with the DPQ Summit Score at 1 year as the dependent variable. All models were adjusted for the baseline DPQ Summit Score. Each predictor was evaluated in a separate model. Continuous variables are reported as β coefficients with 95% CIs, and categorical variables were assessed using joint F tests. To adjust for multiple testing, the Bonferroni correction was applied by multiplying each *P* value by the number of conducted tests, thereby controlling the family-wise error rate. Because each regression model required complete data for both the outcome and the predictor under evaluation, the number of patients contributing to individual models varied. To assess the robustness of the regression analyses to missing data, 2 sensitivity analyses were performed: (1) Baseline characteristics were compared between patients with and without an available 1-year DPQ Summit Score using Student *t* tests and Fisher exact tests, and (2) the regression models were re-estimated after multiple imputation by chained equations (30 multiply imputed datasets), with estimates pooled using Rubin's rules. Assumptions of linear regression were evaluated by inspecting residual normality (histogram, Q–Q plot, Shapiro–Wilk test), homoscedasticity (residual vs fitted plots, Breusch–Pagan test), multicollinearity (variance inflation factors), and influence diagnostics (Cook's distance, leverage, difference in fits). No violations were detected that would compromise model validity. Longitudinal changes were analyzed using linear mixed-effects models with time as a categorical fixed effect (baseline as reference) and a random intercept for each patient to account for within-subject correlation. Pairwise comparisons between time points (baseline vs 3 months, baseline vs 1 year, and 3 months vs 1 year) were estimated using linear contrasts. Results are reported as mean differences with 95% CIs and corresponding *P* values. Statistical analyses were performed using Stata 19 (StataCorp LLC), with a 2-sided significance level of .05.

## Results

Eighty-eight patients had an indication for robotic diaphragm plication during the study period. Five patients were excluded because of intraoperative or clinical findings that precluded study inclusion, but no patients were excluded because of incomplete baseline data. One additional patient was excluded because of missing 30-day follow-up, resulting in a final cohort of 82 patients included between 2019 and 2024 (Figure E1). The mean age was 60.6±10.6 years (Table 1), with a mean body mass index of 30±4.5 kg/m^2^. The majority of the patients were male (73.2%), with 17.1% active smokers. The mean Charlson Comorbidity Index was 0.3±0.6. Baseline pulmonary function showed a restrictive pattern with mean forced vital capacity (FVC) and forced expiratory volume in 1 second (FEV_1_) of approximately 60% predicted. Fluoroscopy demonstrated diaphragm paralysis with paradoxical motion in 86.6% of cases. The etiology was idiopathic in 46%, iatrogenic in 38%, traumatic in 12%, and infectious in 8%.

**Table 1:** Patients' details

Parameter	Mean	SD
Age (y)	60.6	10.6
BMI (kg/m2)	30.1	4.5
ASA status	2.9	0.2
Charlson Comorbidity Index	0.3	0.6
FEV1 (L)	2.1	0.7
FEV1 (%)	60.9	15.2
FVC (L)	2.6	0.8
FVC (%)	60.6	12.7
FEV1/FVC (%)	76.4	10.8
pO2 (mm Hg)	72.0	6.1
DPQ score		
Physical functioning	40.6	18.7
Psychological health	54.0	22.5
Respiratory functioning	24.3	18.6
Social functioning	52.8	21.6
Summit Score	41.9	2.0
Length of symptom (mo)	39.4	52.4
	**n**	**%**
Sex		
Male	60	73.2
Female	22	26.8
Side		
Left	41	50
Right	41	50
Smoking status		
Current	14	17.1
Former	29	35.4
Never	39	47.6
Fluoroscopy		
Paralysis and paradoxical motion	71	86.6
Paralysis without paradoxical motion	6	7.3
Missing	5	6.1
Etiology		
Idiopathic	38	46.3
Infectious	7	8.5
Iatrogenic	27	32.9
Traumatic	10	12.2

*BMI*, Body mass index; *ASA*, American Society of Anesthesiologists physical status classification; *FEV1*, forced expiratory volume in 1 second; *FVC*, forced vital capacity; *DPQ*, Diaphragmatic Paralysis Questionnaire.

The mean length of stay was 5.2 ± 2.6 days, and the mean operative robotic console time was 80 ± 38 minutes (Table 2). Nonabsorbable mesh reinforcement was used in 44 patients (53.7%). The majority of patients experienced an uncomplicated postoperative course during the index hospital stay, with 92.7% of patients showing no in-hospital complications. The most frequent in-hospital event was pleural effusion (2.4%), which was managed conservatively. After discharge, pleural effusion was more frequently observed, occurring in 19.5% of patients, and was treated with diuretics or thoracentesis when necessary. Other postdischarge complications were rare. Overall, according to the Clavien–Dindo classification, the majority of complications were minor (grade I–II), and severe complications (grade ≥ III) were infrequent. One patient experienced a grade IIIb complication (hemothorax) 3 weeks postdischarge, and 2 patients (2.4%) had symptomatic recurrences confirmed by computed tomography. One recurrence occurred after heavy lifting 3 months postoperatively, and the second was idiopathic. Both were successfully revised surgically. At 90 days, 73% of patients reported no thoracic pain, 10% had intermittent pain, and 1% had persistent pain.

**Table 2:** Perioperative details

Parameter	Mean	SD
Length of stay (d)	5.2	2.6
Blood loss (mL)	18.5	15.5
Robotic console time (min)	79.8	38.0
Postoperative drainage time (d)	3.3	2.3
ICU (d)	0.4	0.8
	**n**	**%**
Use of an intraoperative mesh		
Yes	44	53.7
No	38	46.3
In-hospital complications		
Delirium	1	1.2
Pleural effusion	2	2.4
Pulmonary embolism	1	1.2
Pneumonia and hemoptysis	1	1.2
Parenchymal fistula, pneumothorax	1	1.2
No complications	76	92.7
Postdischarge complications		
Wound hematoma (oral anticoagulation)	1	1.2
Pleural effusion	16	19.5
Hemothorax (3 wk after discharge)	1	1.2
Abdominal atony	1	1.2
Pneumonia	1	1.2
Atrial fibrillation	1	1.2
No complications	61	74.4
Clavien-Dindo score		
0	60	73.2
I	9	11.0
II	7	8.5
IIIa	5	6.1
IIIb	1	1.2
Pain 90 d after surgery		
Intermittent	8	9.8
No	60	73.2
Permanent	1	1.2
Missing data	13	15.9
**Recurrence**	2	2.4

*ICU*, Intensive care unit.

Most patients exhibited substantial improvement in respiratory symptoms, including reduced dyspnea and enhanced exercise tolerance (Figure 1 and Table 3). Subsequent follow-ups at 3- and 12-month intervals postsurgery demonstrated sustained improvements in quality of life, as reflected by increased DPQ scores. The median duration of follow-up was 12 months (interquartile range, 3–12; mean 9.4 ± 4.6 months), with 61 of 82 patients (74.4%) completing DPQ assessment at the 1-year time point (Figure E2). Linear mixed-effects models were used to assess longitudinal changes over time.

**Figure 1:**
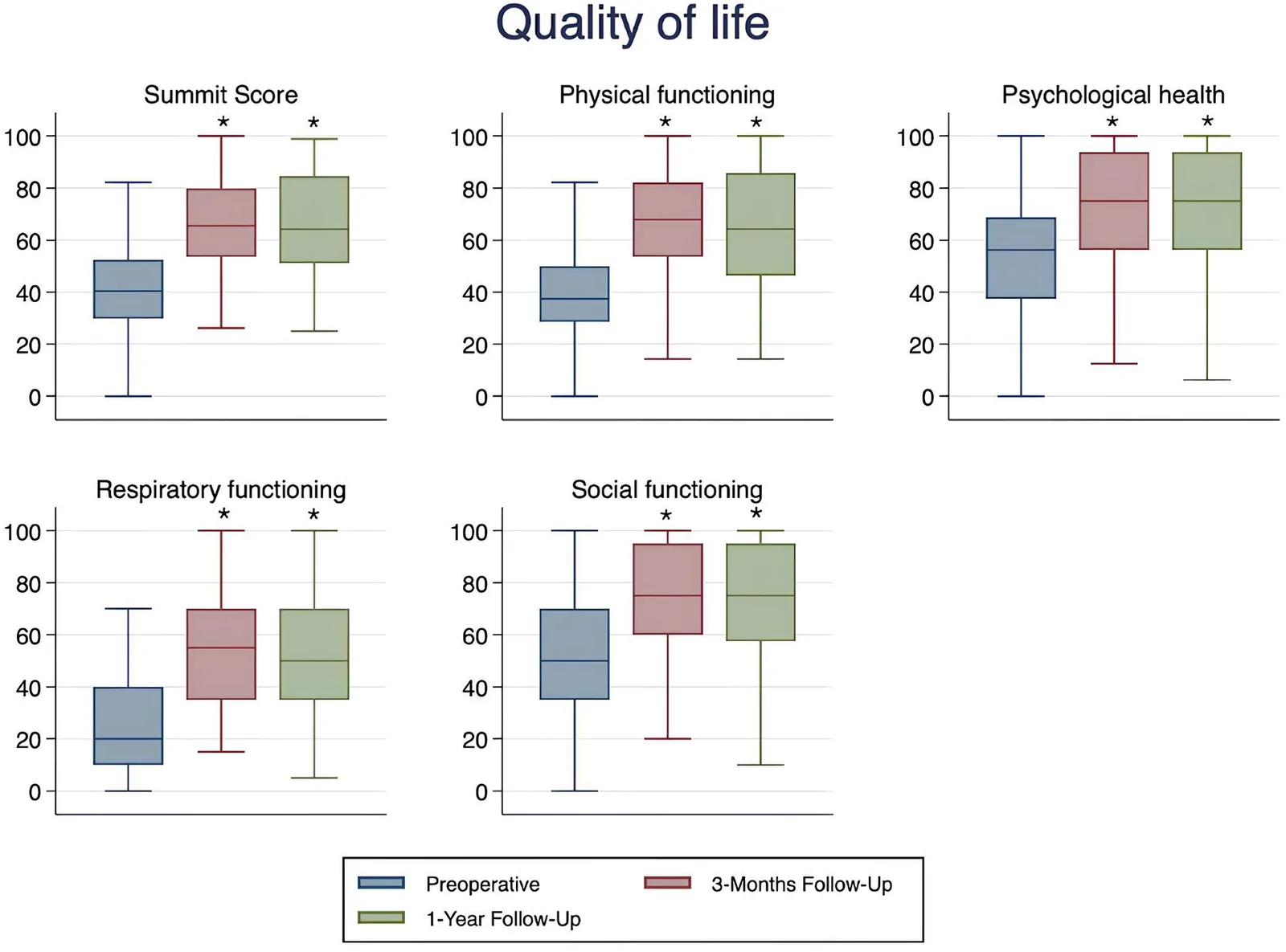
Quality of life after surgery; a total of 82 patients underwent robotic diaphragm plication, with 67 completing the 3-month follow-up and 61 completing the 1-year follow-up. *Boxes* represent the interquartile range (25th-75th percentile), the *horizontal line* within each *box* represents the median, whiskers extend to 1.5 × interquartile range beyond the *box* (or the most extreme nonoutlier value), *dots* indicate outliers beyond that range, and *asterisks* indicate statistically significant differences compared with baseline.

**Table 3:** Quality of life and lung function after surgery

Quality of life score	Values	Mean difference	95% CI	Significance
Social	3 mo vs preoperative	19.75	15.15-24.35	<.001
	1 y vs preoperative	18.27	11.56-24.97	<.001
	1 y vs 3 mo	−1.43	−5.72 to 2.86	.505
Physical	3 mo vs preoperative	26.52	21.49-31.56	<.001
	1 y vs preoperative	27.28	19.60-34.95	<.001
	1 y vs 3 mo	−2.30	−7.40 to 2.79	.366
Psychological	3 mo vs preoperative	17.93	12.48-23.38	<.001
	1 y vs preoperative	13.37	6.34-20.40	<.001
	1 y vs 3 mo	−1.76	−8.07 to 4.55	.577
Respiratory	3 mo vs preoperative	29.34	23.93-34.75	<.001
	1 y vs preoperative	27.38	20.44-34.31	<.001
	1 y vs 3 mo	−4.95	−8.82 to −1.08	.013
Summit	3 mo vs preoperative	24.59	20.21-28.97	<.001
	1 y vs preoperative	23.55	18.58-28.51	<.001
	1 y vs 3 mo	−0.99	−3.91 to 1.93	.499
**Lung function**	**Values**	**Mean Difference**	**95% CI**	**Significance**
FVC (% predicted)	3 mo vs preoperative	9.60	7.33-11.86	<.001
	1 y vs preoperative	9.16	6.45-11.87	<.001
	1 y vs 3 mo	1.06	−1.39 to 3.52	.386
FEV_1_ (% predicted)	3 mo vs preoperative	8.82	6.76-10.88	<.001
	1 y vs preoperative	7.73	5.23-10.23	<.001
	1 y vs 3 mo	−0.15	−2.65 to 2.36	.907
pO_2_	3 mo vs preoperative	0.81	−1.73 to 3.36	.524
	1 y vs preoperative	2.19	−0.34 to 4.73	.088
	1 y vs 3 mo	0.37	−2.33 to 3.07	.783
pCO_2_	3 mo vs preoperative	−0.44	−1.65 to 0.77	.467
	1 y vs preoperative	−0.59	−2.00 to 0.82	.406
	1 y vs 3 mo	0.26	−0.83 to 1.35	.629
DLCO SB (% predicted)	3 mo vs preoperative	3.92	1.04-6.81	.009
	1 y vs preoperative	4.41	−0.30 to 9.13	.066
	1 y vs 3 mo	−1.97	−6.50 to 2.56	.382

Values represent mean differences calculated as follow-up minus preoperative values; positive values indicate improvement. *FVC*, Forced vital capacity; *FEV1*, forced expiratory volume in 1 second; *DLCO SB*, single-breath diffusing capacity of the lung for carbon monoxide.

All DPQ domains improved significantly from baseline to 3 months and remained stable at 1 year. Improvements were observed in the social (mean difference 19.75 and 18.27), physical (mean difference 26.52 and 27.28), psychological (mean difference 17.93 and 13.37), and respiratory domains (mean difference 29.34 and 27.38), as well as in the overall score (mean difference 24.59 and 23.55) (all *P* < .001). Quality of life improvements were largely maintained between 3 months and 1 year, although the respiratory domain showed a small but statistically significant change during this interval (*P* = .013).

Pulmonary function parameters also improved after surgery (Figure 2 and Table 3). FEV_1_ and FVC increased significantly at both 3 months and 1 year (all *P* < .001). Single-breath diffusing capacity of the lung for carbon monoxide showed a modest improvement at 3 months (*P* = .009), which was not sustained at 1 year (*P* = .066). No significant changes were observed in arterial blood gas parameters (partial pressure of oxygen and partial pressure of carbon dioxide).

**Figure 2:**
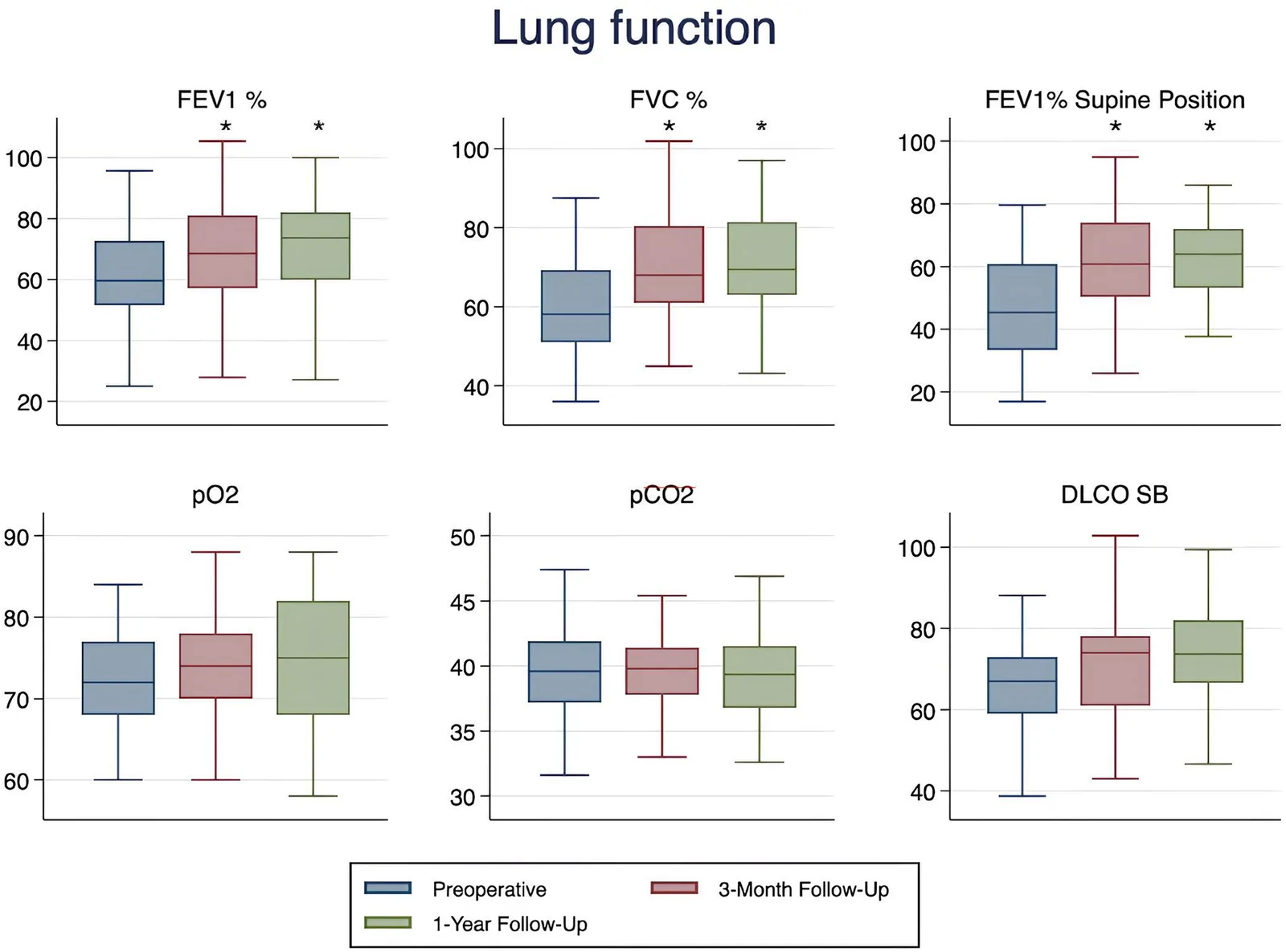
Lung function after surgery; a total of 82 patients underwent robotic diaphragm plication with 67 completing the 3-month follow-up and 61 completed the 1-year follow-up. *Boxes* represent the interquartile range (25th-75th percentile), the *horizontal line* within each box represents the median, whiskers extend to 1.5 × interquartile range beyond the *box* (or the most extreme nonoutlier value), *dots* indicate outliers beyond that range, and *asterisks* indicate statistically significant differences compared with baseline. *FEV1*, Forced expiratory volume in 1 second; *FVC*, forced vital capacity; *DLCO SB*, single-breath diffusing capacity of the lung for carbon monoxide.

In linear regression analyses adjusted for baseline DPQ Summit Score, none of the evaluated continuous predictors were independently associated with 1-year DPQ Summit Score. A borderline association was observed for intraoperative mesh use (overall *P* = .069), whereas baseline lung function, symptom duration, body mass index, age, side, sex, and fluoroscopic sniff test results were not significantly associated with outcome (Table E3).

To assess the potential impact of missing 1-year follow-up data, additional sensitivity analyses were performed. Patients with and without a 1-year DPQ assessment were comparable with respect to most baseline demographic and clinical characteristics (Table E4). However, patients lost to follow-up had lower baseline pulmonary function, with significantly lower FEV_1_, FVC, and Tiffeneau index (% predicted). Re-estimating the regression models after multiple imputation yielded effect sizes and directions consistent with the complete-case analysis, and no predictor reached statistical significance under either approach (fraction of missing information, 0.27–0.39), supporting the robustness of the complete-case analysis.

## Discussion

This study examined long-term quality of life and functional outcomes after minimally invasive robotic-assisted diaphragm plication in patients with symptomatic unilateral diaphragm paralysis. The results suggest that the procedure is safe, associated with minimal perioperative morbidity, and linked to sustained improvements in pulmonary function and patient-reported HRQL.

Our findings expand on previous literature, which has mainly consisted of small retrospective series and limited follow-up data.^9^, ^10^, ^11^, ^12^, ^13^, ^14^, ^15^, ^16^, ^17^, ^18^, ^19^, ^20^, ^21^, ^22^ The present analysis of 82 patients, one of the largest single-center robotic cohorts to date, provides supportive observational evidence regarding the role of robotic-assisted plication as a treatment option for symptomatic unilateral diaphragm paralysis. Previous studies have reported heterogeneous diagnostic pathways and varying attitudes toward surgical intervention in patients with symptomatic unilateral diaphragm paralysis within the chest expert community.^17^ Our experience with these patients demonstrated that they frequently undergo prolonged multidisciplinary diagnostic procedures, resulting in divergent opinions regarding optimal nonsurgical or surgical interventions. The rarity of this disease and the limited number of published surgical series contribute to the lack of evidence regarding long-term treatment outcomes. Additionally, the relationship between objective changes in diaphragm function and objective changes in lung function, as well as subjective health alterations, particularly health-related quality of life, is unclear.

The present study is the first to systematically evaluate HRQL using the DPQ, a validated instrument specifically designed for this patient population.^12^ The use of the DPQ represents a key methodological strength of this study. Unlike generic quality of life instruments such as the SF-36^23^ or respiratory-specific tools such as the SGRQ^24^ or the MRC dyspnea scale,^25^ the DPQ is specifically tailored to diaphragmatic dysfunction. It captures not only respiratory symptoms but also physical limitations, psychological burden, and social impact, which are highly relevant in this patient population. This disease-specific approach may allow more sensitive detection of clinically meaningful postoperative effects that may not be fully captured by more general instruments. This is particularly relevant in diaphragmatic paralysis, where symptom burden extends beyond respiratory impairment alone. We found a significant improvement in the DPQ Summit Score 1 year after surgery, representing a clinically meaningful benefit. Improvements were observed across all DPQ domains and were largely maintained throughout the first postoperative year. Patients reported reductions in dyspnea and enhanced exercise tolerance, consistent with objective gains in FEV_1_. The overall improvement observed across DPQ domains suggests a relevant clinical benefit for patients, supporting the role of diaphragm plication in improving daily functioning and symptom burden. These findings highlight the importance of incorporating disease-specific patient-reported outcomes when evaluating surgical interventions in this population.

Interestingly, arterial blood gas parameters remained unchanged postoperatively, whereas improvements in DLCO were modest and not sustained at 1 year. This is physiologically plausible because most patients did not exhibit baseline gas exchange abnormalities at rest. Future studies incorporating exercise testing could better capture postoperative improvements in dynamic respiratory performance.

Quality of life can be significantly impaired by postoperative complications or chronic pain after surgery. Consequently, an excellent postoperative outcome in terms of lung function can be negatively affected by surgical side effects. Therefore, the overall outcome crucially depends on the invasiveness and safety of the surgical procedure. The positive effects on quality of life and the low complication rate observed after robotic plication in this study cannot be compared with other surgical approaches, because these were not performed at our institution, which represents a limitation of the study.

The monocentric study demonstrated that robotic-assisted thoracoscopic diaphragm plication is a safe procedure with a short length of stay and little risk of severe complications. Despite the overall positive outcomes, some patients did experience complications. Pleural effusion, the most common event, was self-limiting or successfully managed with conservative therapy. Diaphragm plication may alter intrathoracic dynamics and contribute to postoperative pleural fluid accumulation. This underscores the necessity for meticulous postoperative monitoring and management to avert potential risks. Only 1 patient required reoperation for hemothorax, confirming the overall safety of the procedure. The low rate of persistent pain (1%) is noteworthy and may reflect the minimally invasive nature of the robotic approach, reduced intercostal trauma, and effective pain control protocols.

In regression analyses adjusted for baseline quality of life, no preoperative clinical or functional variable independently predicted the DPQ Summit Score at 1 year. Baseline quality of life emerged as the dominant determinant of postoperative outcomes, consistent with ceiling effects and regression toward the mean. Despite significant group-level improvements in pulmonary function, baseline lung function and changes in lung function were not associated with individual patient-reported outcomes, underscoring the limited prognostic value of physiological measures for long-term quality of life. Different reinforcement strategies have been described in the literature, including both permanent and absorbable mesh materials. Absorbable reinforcement may theoretically reduce the amount of permanent foreign material, whereas permanent mesh may provide more durable structural support.^8^^,^^26^ However, comparative evidence between approaches remains limited, and no clear superiority of one strategy over another has been established. Overall, these findings suggest that although surgical intervention results in substantial and sustained improvements in quality of life, interindividual variability in long-term patient-reported outcomes is not well explained by traditional demographic or functional parameters, highlighting the need to consider additional determinants of postoperative recovery.

### Limitations

This study has several limitations. First, despite prospective data collection, the analysis was retrospective, and no control group was included for comparison with other surgical techniques or conservative management. Second, approximately 25% of patients did not complete the 1-year follow-up, which could introduce attrition bias. Patients lost to follow-up had modestly poorer baseline pulmonary function than those who completed follow-up, suggesting that missingness was not entirely random. Consequently, the estimated 1-year treatment effect may have been influenced, although the direction and magnitude of any potential bias cannot be determined. Patients with poorer baseline pulmonary function may have been underrepresented at 1 year, potentially leading to either a slight overestimation of postoperative outcomes or, conversely, an underestimation if these patients had greater potential for postoperative improvement. Nevertheless, sensitivity analyses, including comparisons between completers and noncompleters and multiple-imputation analyses, yielded findings consistent with the primary analyses, supporting the robustness of the reported associations. However, the modest sample size and incomplete follow-up may still have limited the precision of the regression analyses, which should therefore be considered exploratory and hypothesis-generating rather than confirmatory. Third, the study was conducted at a single high-volume tertiary center with substantial robotic experience, potentially limiting generalizability. Furthermore, quality of life assessment was limited to 1 year; longer-term follow-up is warranted to evaluate the durability of functional benefits. Finally, although the DPQ represents a disease-specific instrument for diaphragmatic dysfunction, its initial validation was based on a relatively small patient cohort, and comparative performance against broader HRQL instruments such as the SF-36, SGRQ, or MRC has not yet been established. In addition, the absence of comparator groups precludes conclusions regarding the relative value of robotic-assisted plication compared with alternative surgical approaches.

## Conclusions

Despite these limitations, the present study provides supportive observational evidence that robotic thoracoscopic diaphragm plication is associated with improvements in both objective and subjective outcomes (Figure 3). The combination of a standardized surgical approach, validated HRQL assessment, and systematic data collection represents a methodological strength rarely achieved in this field.

**Figure 3:**
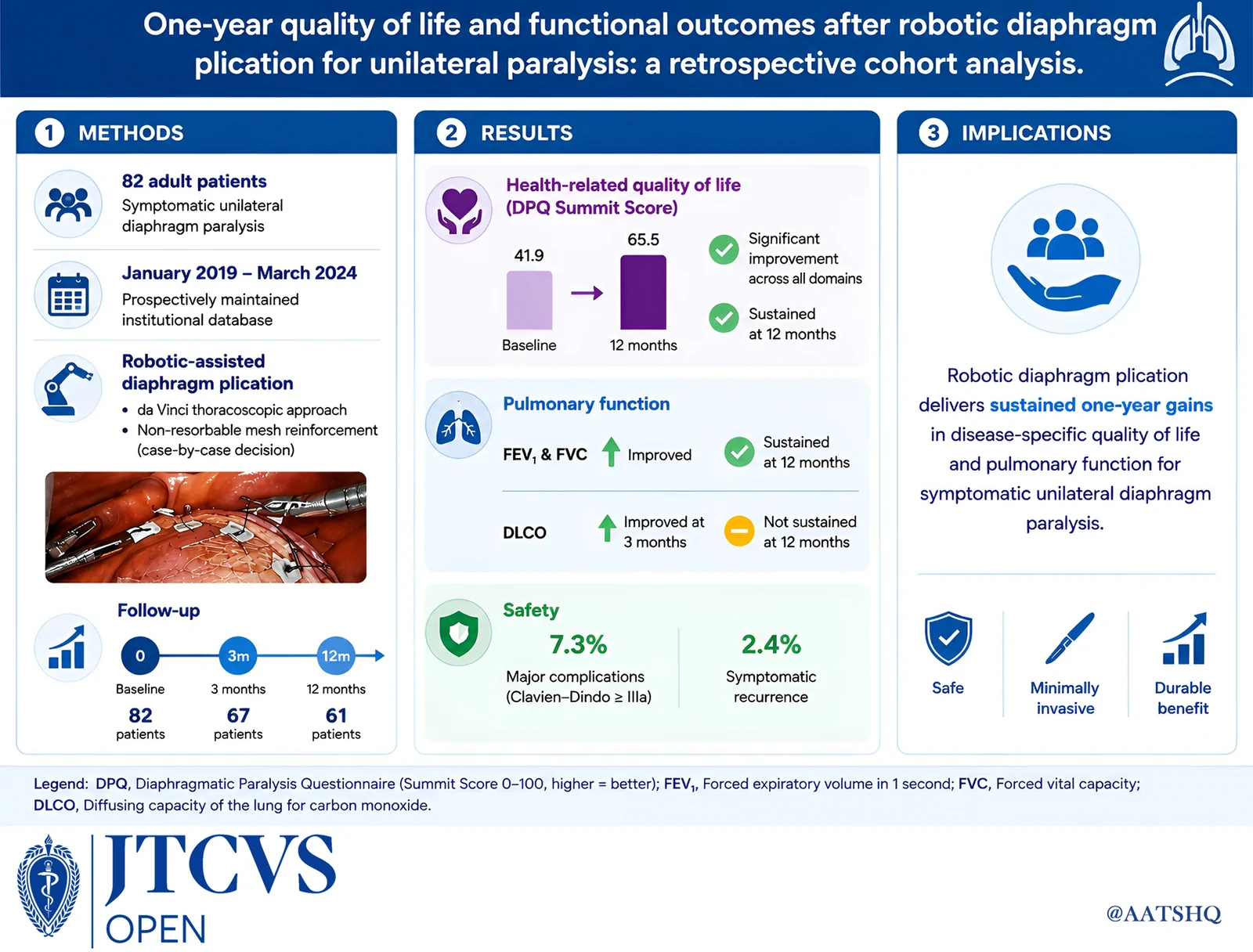
Graphical abstract summarizing study design and main clinical outcomes of robotic diaphragm plication, showing improvements in quality of life and pulmonary function at 12 months.

Our findings suggest that robotic-assisted thoracoscopic diaphragm plication is associated with improved quality of life and respiratory function in patients with symptomatic unilateral diaphragm paralysis. Future studies are needed to further elucidate the long-term benefits and potential risks associated with this surgical approach, as well as further refine standardized diagnostic and therapeutic pathways and reduce unnecessary investigations for this disease.

## Data Availability

The data underlying this article will be shared on reasonable request to the corresponding author.

## Conflict of Interest Statement

M.E.E. and H.W. have received consulting fees from Intuitive Surgical and serve as proctors for the company. M.S. has received honoraria for lectures from VitalAire, Löwenstein Medical, and Novamed. All other authors declare no potential conflicts of interest.

The *Journal* policy requires editors and reviewers to disclose conflicts of interest and to decline handling or reviewing manuscripts for which they may have a conflict of interest. The editors and reviewers of this article have no conflicts of interest.

## Figures and Tables

**Figure E1:**
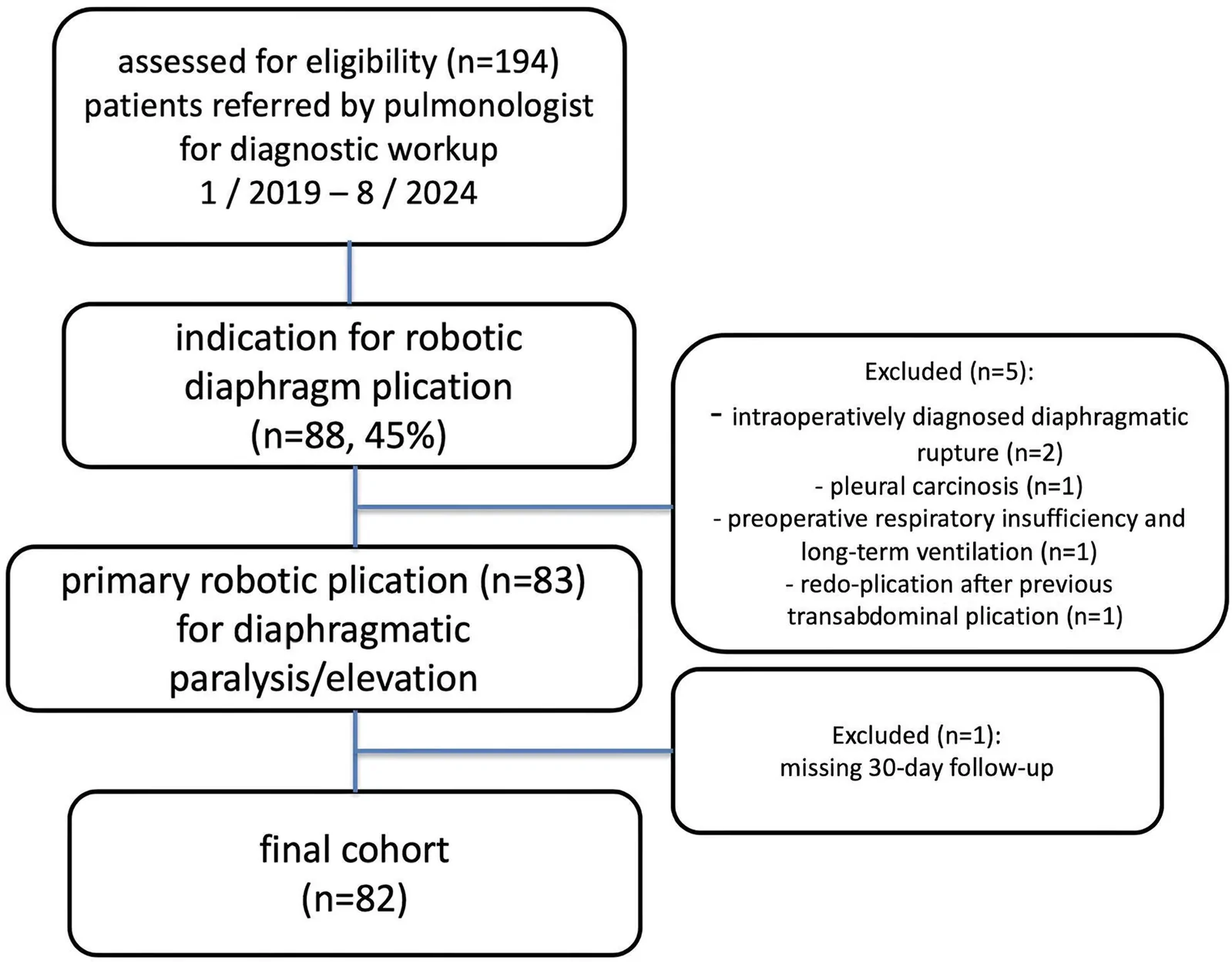
Flow diagram of patient selection and final cohort (n = 82).

**Figure E2:**
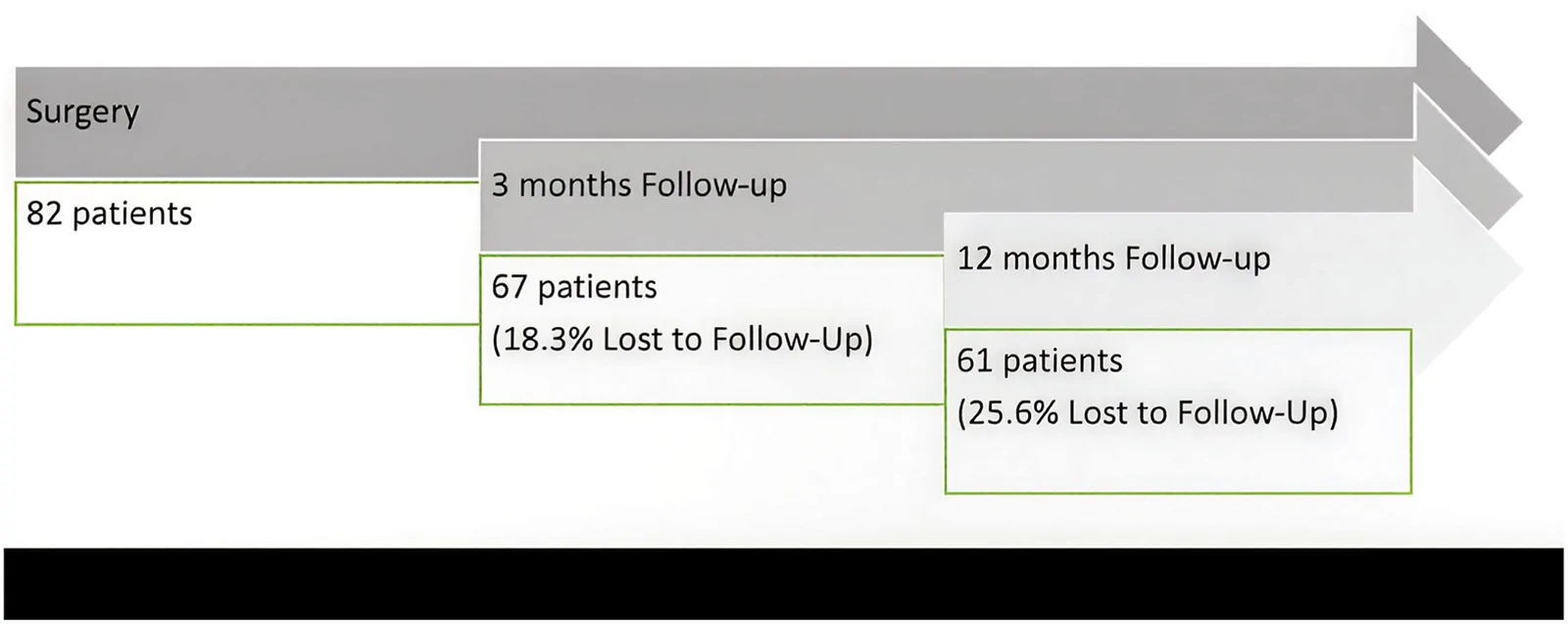
Patient follow-up flow diagram showing follow-up completeness at 3 and 12 months after surgery.

**Table E1:** Summary of reported minimally invasive diaphragm plication series

Study	y	Character	N	Approach	Mortality	Follow-up	Length of stay (d)	Surgery time	Method
Video-assisted surgery									
Demos et al^9^	2008-2015	retrospective	18	thoracoscopic	0%	Median 29.4 mo	Median 3	unknown	mattress suture
Freeman^10^ (11 thoraco-tomies)	2001-2007	prospective	41	Thoracoscopic (n = 30)	0%	Mean 48 mo	Mean 3.7	unknown	mattress suture
Kocher^18^	2015-2016	retrospective	6	thoracoscopic	0%	Mean 3 mo	Mean 3.2	Mean 67 min	stapling of abundant diaphragm
Nardini^15^^,^∗	2014-2018	prospective	35	thoracoscopic	0%	Mean 6-12 mo	Mean 5.9	unknown	mattress suture
Mouroux^19^	1992-2003	prospective	12	thoracoscopic	0%	Mean 64.4 mo	Mean 3.4	unknown	mattress suture
Yalcinkayal^20^	2009-2015	retrospective	37	thoracoscopic	0%	Mean 19 mo	Mean 3.1	Mean 49 min	mattress suture
Taberhaml^21^	2006-2016	retrospective	22	thoracoscopic	0%	none	Mean 4.2	unknown	mattress suture
Beshai^12^ (46 thoraco-tomies/88 thoracoscopic)	2008-2022	retrospective	94	Thoracoscopic (n = 88)	0%	Median 24 mo	Median 5	unknown	mattress suture
Robotic-assisted surgery									
Bin Asaf^16^	2012-2018	retrospective	12	abdominal	0%	6 mo		Mean 242 min	mattress suture with onlay mesh
		retrospective	6	thoracoscopic	0%				
Nardi^15^^,^∗	2014-2018	prospective	14	thoracoscopic	0%	6-12 mo	Mean 5.9	unknown	mattress suture
Le^22^^,^†	2015-2022	retrospective	31	thoracoscopic	0%	Median 12 Months	unknown	Median 109 min	mattress suture

∗ Study includes VATS and RATS cases.

† Additional 12 VATS cases are not shown in this table.

**Table E2:** STROBE Statement—Checklist of items that should be included in reports of cohort studies

Parameter	Item No	Recommendation	Page/Line
Title and abstract	1	(a) Indicate the study's design with a commonly used term in the title or the abstract	1/2
		(b) Provide in the abstract an informative and balanced summary of what was done and what was found	3/45-51
Introduction			
Background/rationale	2	Explain the scientific background and rationale for the investigation being reported	6/81-101
Objectives	3	State specific objectives, including any prespecified hypotheses	6/101-104
Methods			
Study design	4	Present key elements of study design early in the paper	7/107-115
Setting	5	Describe the setting, locations, and relevant dates, including periods of recruitment, exposure, follow-up, and data collection	7-8/107-150
Participants	6	(a) Give the eligibility criteria, and the sources and methods of selection of participants. Describe methods of follow-up	7/116-122; 8/143-149
		(b) For matched studies, give matching criteria and number of exposed and unexposed	N/A
Variables	7	Clearly define all outcomes, exposures, predictors, potential confounders, and effect modifiers. Give diagnostic criteria, if applicable	7/116-122; 8/146-149
Data sources/measurement	8∗	For each variable of interest, give sources of data and details of methods of assessment (measurement). Describe comparability of assessment methods if there is more than one group	*8-9/150-166*
Bias	9	Describe any efforts to address potential sources of bias	*8-9/150-166*
Study size	10	Explain how the study size was arrived at	7/116-122; 8/143-149
Quantitative variables	11	Explain how quantitative variables were handled in the analyses. If applicable, describe which groupings were chosen and why	*8-9/150-166*
Statistical methods	12	(a) Describe all statistical methods, including those used to control for confounding	*8-9/150-166*
		(b) Describe any methods used to examine subgroups and interactions	*8-9/150-166*
		(c) Explain how missing data were addressed	*8-9/150-166*
		(d) If applicable, explain how loss to follow-up was addressed	*8-9/150-166*
		(e) Describe any sensitivity analyses	N/A
Results			
Participants	13∗	(a) Report numbers of individuals at each stage of study—eg numbers potentially eligible, examined for eligibility, confirmed eligible, included in the study, completing follow-up, and analysed	9-10/168-212
		(b) Give reasons for non-participation at each stage	9-10/168-212
		(c) Consider use of a flow diagram	Figure E1
Descriptive data	14∗	(a) Give characteristics of study participants (eg demographic, clinical, social) and information on exposures and potential confounders	9-10/168-212 + Table 1
		(b) Indicate number of participants with missing data for each variable of interest	9/189-190 + Figure E1
		(c) Summarise follow-up time (eg, average and total amount)	9/187-189 + Figure E1
Outcome data	15∗	Report numbers of outcome events or summary measures over time	Table 2 and 3 - Figure 1 and 2
Main results	16	(a) Give unadjusted estimates and, if applicable, confounder-adjusted estimates and their precision (eg, 95% confidence interval). Make clear which confounders were adjusted for and why they were included	9-10/168-212 + Table 3
		(b) Report category boundaries when continuous variables were categorized	N/A
		(c) If relevant, consider translating estimates of relative risk into absolute risk for a meaningful time period	N/A
Other analyses	17	Report other analyses done—eg analyses of subgroups and interactions, and sensitivity analyses	10/208-212
Discussion			
Key results	18	Summarise key results with reference to study objectives	10-11/215-219
Limitations	19	Discuss limitations of the study, taking into account sources of potential bias or imprecision. Discuss both direction and magnitude of any potential bias	13/281-287
I nterpretation	20	Give a cautious overall interpretation of results considering objectives, limitations, multiplicity of analyses, results from similar studies, and other relevant evidence	11-13/220-280
Generalisability	21	Discuss the generalisability (external validity) of the study results	13/288-297
Other information			
Funding	22	Give the source of funding and the role of the funders for the present study and, if applicable, for the original study on which the present article is based	14/306-308

An Explanation and Elaboration article discusses each checklist item and gives methodological background and published examples of transparent reporting. The STROBE checklist is best used in conjunction with this article (freely available on the Web sites of PLoS Medicine at http://www.plosmedicine.org/, Annals of Internal Medicine at http://www.annals.org/, and Epidemiology at http://www.epidem.com/). Information on the STROBE Initiative is available at http://www.strobe-statement.org.

∗ Give information separately for exposed and unexposed groups.

**Table E3:** Predictors of 1-year DPQ Summit Score

Predictor	β coefficients	Significance	n	R^2^
FEV1	4.40 (−2.23 to 11.02)	0.199	57	.31058844
Δ FEV1 (upright)	−4.97 (−17.99 to 8.06)	0.458	53	.31893015
Length of symptoms	−0.00 (−0.00 to 0.00)	0.840	55	.34500355
BMI	−0.46 (−1.38 to 0.46)	0.333	57	.30133894
Tiffenau-Index	0.33 (−0.12 to 0.79)	0.157	57	.31513071
Age	−0.07 (−0.52 to 0.37)	0.744	57	.29038839
Intraoperative use of Mesh	—	0.069	57	.3317007
Sniff-Test (Paradox Movement)	—	0.228	57	.32752236
Side	—	0.077	57	.32937837
Sex	—	0.949	57	.28903291

β coefficients from linear regression were adjusted for baseline DPQ Summit Score. *FEV*_*1*_, Forced expiratory volume in one second; *BMI*, body mass index.

**Table E4:** Comparison of baseline characteristics between patients who completed the 1-year follow-up and those lost to follow-up

Variable	Completed 1-y follow-up (n = 61)	Lost to follow-up (n = 21)	*P*-value
Age (y)	59.6 ± 10.4	63.8 ± 11.0	.129
ASA status	3.0 ± 0.13	3.0 ± 0.22	.541
Charlson Comorbidity Index	0.28 ± 0.64	0.19 ± 0.40	.786
FEV_1_ (L)	2.13 ± 0.71	1.89 ± 0.52	.104
FEV_1_ (% predicted)	63.0 ± 15.7	54.9 ± 12.3	.019
FVC (% predicted)	62.4 ± 13.0	55.7 ± 10.4	.022
Tiffeneau index (%)	77.9 ± 10.9	72.2 ± 9.3	.026
DLCO (% predicted)	67.6 ± 12.6	61.1 ± 13.3	.060
pO_2_ (mm Hg)	72.5 ± 5.8	71.3 ± 7.2	.517
Sex (female)	19 (31.2%)	3 (14.3%)	.163
Side (left)	31 (50.8%)	10 (47.6%)	1.000
Smoking Status			
Current	10 (16.4%)	4 (19.1%)	.986
Former	21 (34.3%)	8 (38.1%)	
Never	30 (49.2%)	9 (42.9%)	
Etiology			
Idiopathic	27 (44.3%)	11 (52.4%)	.477
Infectious	6 (9.8%)	1 (4.8%)	
Iatrogenic	22 (36.1%)	5 (23.8%)	
Traumatic	6 (9.8%)	4 (19.1%)	
Length of stay (d)	4.9 ± 2.2	6.3 ± 3.4	.088

Data are presented as mean ± standard deviation or n (%). Continuous variables were compared using Welch's two-sample *t*-test (unequal variances); the Charlson Comorbidity Index was compared using the Mann-Whitney *U* test; categorical variables were compared using Fisher's exact test. Completion of the 1-year follow-up was defined by availability of the 1-year Diaphragm Plication Questionnaire (DPQ) summit score; where the 1-year assessment was unavailable, the 2-year value was carried forward.*n*, number of patients; *ASA*, American Society of Anesthesiologists physical status classification; *FEV*_*1*_, forced expiratory volume in 1 s; *FVC*, forced vital capacity; *DLCO*, single-breath diffusing capacity of the lung for carbon monoxide; *pO*_*2*_, partial pressure of oxygen.
